# Wearable Loop Sensor for Bilateral Knee Flexion Monitoring

**DOI:** 10.3390/s24051549

**Published:** 2024-02-28

**Authors:** Yingzhe Zhang, Jaclyn B. Caccese, Asimina Kiourti

**Affiliations:** 1 ElectroScience Laboratory, Department of Electrical and Computer Engineering, The Ohio State University, Columbus, OH 43212, USA; zhang.12524@osu.edu; 2 School of Health and Rehabilitation Sciences, The Ohio State University, Columbus, OH 43210, USA; jaclyn.caccese@osumc.edu

**Keywords:** wearable sensor, joint flexion, bioelectromagnetic, Faraday’s law, electromotive force, e-textiles

## Abstract

We have previously reported wearable loop sensors that can accurately monitor knee flexion with unique merits over the state of the art. However, validation to date has been limited to single-leg configurations, discrete flexion angles, and in vitro (phantom-based) experiments. In this work, we take a major step forward to explore the bilateral monitoring of knee flexion angles, in a continuous manner, in vivo. The manuscript provides the theoretical framework of bilateral sensor operation and reports a detailed error analysis that has not been previously reported for wearable loop sensors. This includes the flatness of calibration curves that limits resolution at small angles (such as during walking) as well as the presence of motional electromotive force (EMF) noise at high angular velocities (such as during running). A novel fabrication method for flexible and mechanically robust loops is also introduced. Electromagnetic simulations and phantom-based experimental studies optimize the setup and evaluate feasibility. Proof-of-concept in vivo validation is then conducted for a human subject performing three activities (walking, brisk walking, and running), each lasting 30 s and repeated three times. The results demonstrate a promising root mean square error (RMSE) of less than 3° in most cases.

## 1. Introduction

Monitoring human kinematics in real-world environments is becoming increasingly important in diverse fields, such as healthcare (e.g., prevention [1], rehabilitation [2,3,4], training [3]), sports (e.g., performance analysis and optimization [5,6,7], energy estimation [8]) and virtual reality [9,10,11]. As an example, athletes suffer from an elevated risk of subsequent mild traumatic brain injury (mTBI) and musculoskeletal injury after recovery from an initial mTBI [12,13]. This susceptibility is predominantly attributed to postural control impairments, which serves as both diagnostic criteria and prognostic indicators for mTBI and prolonged recovery [12,14,15]. However, the gap between the increased risk and postural control impairments remains unclear because of the inability to effectively analyze and track postural control during actual athletic activities after recovery [12,13,16,17]. This practice raises concerns that athletes might be admitted returning to play while still suffering from undetected impairments, increasing the risk of subsequent injuries.

In turn, wearable sensors capable of monitoring human kinematics in uncontrived settings, seamlessly, and with high accuracy, entail an emerging research area. Typically, postural control assessment employs sophisticated laboratory equipment, such as optoelectronic motion capture (MoCap) systems [3,11,18,19], to yield detailed quantitative data. MoCap systems are highly accurate and used as today’s “gold-standard”, but they are confined to constrained environments. Though wearable motion sensors have been reported in the state of the art, they suffer from inherent limitations. Referring to Table 1, state-of-the-art wearable motion capture sensing approaches can be categorized as either direct or indirect.

- Direct methods place sensors directly on the joint and measure the angle utilizing flexible materials. Examples include fiber-optic sensors and bending sensors. Fiber-optic sensors [20,21,22], while capable of bending along with the joint, do not stretch very well, hampering the individual’s natural movement. Bending sensors, on the other hand, are both bendable and stretchable. Nevertheless, resistive-type bending sensors are prone to hysteresis from stretching and bending deformations [23,24,25,26], while capacitive-based bending sensors may encounter errors due to capacitive coupling when skin contacts the sensors, potentially compromising accuracy [27,28]. Additionally, the direct placement of these sensors upon the joints makes them uncomfortable and somewhat limits movement.
- Indirect methods rely on computing the relative position of sensors placed on limb parts adjacent to the joint, but not directly on the joint (e.g., to monitor knee angles, sensors are placed on the shank and thigh, respectively, as opposed to the knee joint itself). By doing so, indirect methods eliminate the discomfort and movement restrictions associated with the abovementioned direct methods. Technologies such as inertial measurement units (IMUs) and time-of-flight sensors fall into this category. IMUs, which combine an accelerometer, gyroscope, and magnetometer in a miniaturized unit, and derive angles by dual integration of raw acceleration measurements [5,7,8,22,29,30,31,32]. However, they necessitate sophisticated signal processing methods (e.g., Kalman filtering), require continuous calibration to address the integration drift, and are known to be bulky and injury prone. Time-of-flight sensors, typically based on electromagnetic/radar [32,33] or ultrasound [34,35] operating principles to measure the distance between the units, are highly susceptible to electromagnetic and acoustic interference.

To overcome the limitations, we recently proposed a new class of electromagnetics-based wearable loop sensors that are reliable, cost-effective, and comfortable, specifically designed for use in non-contrived settings [36,37]. With a focus on sagittal knee flexion, the sensor comprises two loops symmetrically placed on the shank and thigh that operate in the inductive regime and act as transmitter and receiver, respectively. Joint flexion causes the loops to misalign and, based on Faraday’s law, alters the voltage on the receiving loop. However, the validation of our sensor to date has been limited to the static angles of a single leg as tested upon a tissue-emulating phantom and benchmarked against a goniometer—a method lacking in precision. 

In this paper, we take a major step forward by monitoring real-time sagittal flexion angles, on both legs (bilateral), on human subjects, as validated against a “gold-standard” camera-based motion capture system. Novelty of this work lies in:

1. A wearable loop-based sensor configuration for bilateral knee flexion monitoring. This is the first time that we explore the feasibility of placing wearable loop sensors on both legs, concurrently. We report a theoretical analysis that addresses aspects related to interference, propose a bilateral sensor setup, and study the resulting angular resolution for each of the legs.
2. A new fabrication process that enhances the stability of the connection between the loops and the SMA connector used for signal transmission/reception. The proposed approach utilizes a creative combination of conductive threads (e-threads) and polymer materials to ensure flexibility, comfort, and mechanical robustness. Besides the loop sensors of this study, the proposed fabrication approach is relevant to several flexible electronics applications.
3. An analysis of the sensor’s sources of error with a goal to further optimize the design in the future. Besides anticipated sources of external noise (e.g., loop deformation, loop misalignment, ambient noise), we introduce and analyze a new source of internal noise that relates to the presence of motion electromotive force (EMF). Notably, the abovementioned sources of error are confirmed and quantified in realistic settings (i.e., for a human subject performing dynamic activities).
4. Demonstration of superior angular accuracy vs. prior wearable sensing approaches (see root mean square error, RMSE, values in Table 1), as validated upon a human subject performing dynamic activities.

**Table 1:** Comparison of state-of-the-art wearable sensors for joint kinematics monitoring.

		Lightweight	Insensitive to Environment Noise	Allows Natural Motion	Easy to Implement	RMSE Values (Type of Activity Performed)
Direct	Optic-based [20]	Yes	Yes	No	Yes	5.31° ± 1.13° (5 bending cycles × 10 participants)
	Bending (resistive) [25]	Yes	Yes	No	Yes	<5° (walking for 60 s × 3 participants) <15° (running for 60 s × 3 participants)
	Bending (capacitive) [28]	Yes	No	No	Yes	5.8° ± 1.2° (robotic arm bending for 10 min)
Mix of Direct and Indirect	IMU + Optic-based [22]	No	Yes	No	No	3.28° ± 1.12° (20 gait cycles × 12 participants))
Indirect	IMU [31]	Yes	Yes	Yes	No	3.3° (walking for 10 s)
	IMU + radar [32]	No	Yes	Yes	No	3.5° ± 1.1° (walking for 100 s × 4 times) 4.5° ± 1.3° (running for 100 s × 4 times)
	Radar [33]	No	No	No	Yes	0 to 7.2° (with increasing angular speed from 0 to 150 deg/s for 1 min × 10 times)
	Wearable loop sensors (this work)	Yes	Yes	Yes	Yes	<3.2° (walking, brisk walking and running for 30 s × 3 times per motion)

## 2. Materials and Methods

In this section, we commence with a theoretical analysis of the proposed bilateral sensor system (Section 2.1). We first evaluate feasibility and lay down the equations of operation based on Faraday’s law of induction (Section 2.1.1). Unlike configurations with two loops for a single leg, interference among the four loops must now be small enough to avoid ambiguities (Section 2.1.2). A comprehensive noise analysis further sets expectations for increased errors at small angles and higher motion speeds, with particular focus on motion EMF, reported herewith for the first time (Section 2.1.3). Methodologies to evaluate the sensor numerically and experimentally are then outlined in Section 2.3. Of particular interest is a novel fabrication methodology for polymer-embedded e-thread-based loops that ensure flexibility and conformality along with mechanical robustness (Section 2.3.1). 

### 2.1. Theoretical Background

#### 2.1.1. Bilateral Sensor Operation 

**Figure 1:**
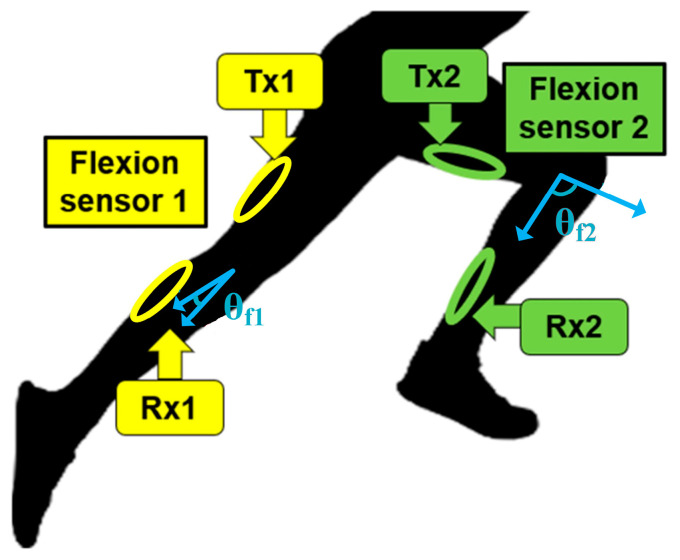
Proposed wearable loop sensors for monitoring bilateral knee flexion angles by capturing *θ_f_*_1_ and *θ_f_*_2_ from two receiving loops, *Rx*1 and *Rx*2, respectively.

As illustrated in Figure 1, the proposed wearable sensor for bilateral knee flexion monitoring comprises four loops: two act as transmitters (*Tx*1 and *Tx*2) and are placed on the thighs, and two serve as receivers (*Rx*1 and *Rx*2) and are placed on the shanks. Each *Tx* and the corresponding *Rx* are positioned symmetrically relative to the knee joint. These four loops all resonate in the deep induction region for reasons outlined in our previous work [36]. If we regard the loops on both legs as independent, the induced voltage on *Rx*1 and *Rx*2, based on Faraday’s law of induction, can be expressed as:

(1) $$V_{Rx1}=-\frac{d}{dt}\iint \vec{B_{Tx1}}\cdot \hat{n_{Rx1}}\;ds_{1}=-\frac{d}{dt}\iint \left|\vec{B_{Tx1}}\right|\cdot cos\theta_{f1}ds_{1}$$

(2) $$V_{Rx2}=-\frac{d}{dt}\iint \vec{B_{Tx2}}\cdot \hat{n_{Rx2}}\;ds_{2}=-\frac{d}{dt}\iint \left|\vec{B_{Tx2}}\right|\cdot cos\theta_{f2}ds_{2}$$

where $B_{Tx1}$ and $B_{Tx2}$ represent the magnetic field density of *Tx*1 and *Tx*2, $\hat{n_{Rx1}}$ and $\hat{n_{Rx2}}$ are the unit vectors normal to *Rx*1 and *Rx*2, $s_{1}$ and $s_{2}$ represent the surface integral regions of *Rx*1 and *Rx*2, respectively, and *θ_f_*_1_ and *θ_f_*_2_ represent the flexion angle of the joints (per definition in Figure 1).

**Figure 2:**
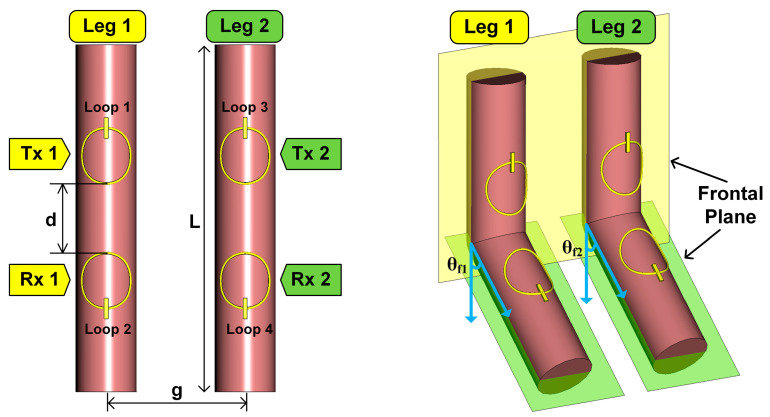
Simulation setup for the wearable loop sensor placed on cylindrical limb model for bilateral knee flexion angle monitoring.

Joint flexion alters the relative position of the *Tx* and *Rx* loops on each leg, in turn altering the direction of the unit vector on each of the *Rx* loops ($\hat{n_{Rx1}}$ and $\hat{n_{Rx2}}$). Per Equations (1) and (2), this change in angle between the unit vectors and magnetic fields is reflected as change in the joint flexion angles, *θ_f_*_1_ and *θ_f_*_2_. This relationship establishes a foundational link between the induced voltages on *Rx*1 and *Rx*2 (or, equivalently, the transmission coefficient parameters between the loops, |S_21_| and |S_43_|, per loop numbering in Figure 2) and the respective flexion angles. This enables the deduction of *θ_f_*_1_ and *θ_f_*_2_ from these induced voltages, or, equivalently, from |S_21_| and |S_43_|.

#### 2.1.2. Electromagnetic Interference Analysis 

The introduction of two additional loops to monitor flexion on the second limb necessitates a thorough consideration of potential interference issues. Specifically, the single-leg monitoring configuration could be modeled as two serial RLC resonant circuits. Now, the addition of two more loops is equivalent to including two additional serial RLC circuits into the system. Notably, mutual inductance considerations may now alter the equivalent impedance of each loop. For example, if *θ_f_*_1_ remains constant and *θ_f_*_2_ varies, the equivalent impedances of *Tx*1 and *Tx*2 change, leading to possible voltage changes in *Rx*1. Ambiguity arises when the mutual inductance value is substantial enough to significantly alter the equivalent impedance. We hypothesize that the proposed wearable sensor is prone to negligible mutual inductance issues, such that there is minimal interference among loops from each leg. This hypothesis is validated numerically and experimentally in Section 3.1.1.

#### 2.1.3. Sources of Noise 

Conducting a comprehensive error analysis is critical for understanding the sensor operation and for guiding future design improvements. In this section, we discuss possible sources of error, herewith classified as:

- External noise: The one-to-one relationship between transmission coefficient (|S_21_| and |S_43_|) and angle (*θ_f_*_1_ and *θ_f_*_2_) described in Section 2.1.1 is expected to be susceptible to noise caused by external factors, such as loop deformation, loop misalignment, and ambient noise. In previous work [36], we highlighted that the abovementioned relationship (also known as calibration curve) becomes notably flat at smaller flexion angles. As such, we expect loop movement during dynamic motion as well as fluctuations of S-parameter values caused by ambient noise to reduce the sensor’s accuracy at smaller flexion angles. In turn, this necessitates sensor design with sharp (i.e., high slope) calibration curves.
- Internal noise: Internal noise primarily originates from motion EMF, an inherent error caused by unwanted voltage induced in moving objects. Notably, this is the first time that motion EMF is discussed for wearable loop sensors: our prior analysis [36,37], which assumed the *Rx* loop to be stationary, did not account for motion EMF. Nevertheless, as the joint undergoes flexion and extension, the relative position of the *Rx* loops changes, necessitating a more comprehensive formula to accurately express the induced voltage. This expanded formulation, as depicted in Equation (3), takes into account the dynamic nature of the *Rx* loop’s position, offering a more accurate model for the induced voltage under real movement conditions. Specifically, the total induced voltage on the loop is:

(3) $$V_{ind}=\frac{d\emptyset \left(\vec{r_{0}}\right)}{dt}+\frac{d\emptyset \left(\vec{r}\right)}{dt}$$

where $\vec{r_{0}}$ represents the initial spatial vector, $\vec{r}$ represents the relative position vector from the initial point, θ is the flexion angle of the joint, and ∅ represents the magnetic flux. 

Specifically, Equation (3) delineates two distinct components of induced voltage. The first term corresponds to the induced voltage resulting from time-varying current in a stationary loop. Conversely, the second term introduces the induced volage attributed to the loop’s spatial movement. Within our sensor model, the spatial movement vector ($\vec{r})$ can be approximated by the flexion angle (θ), as shown in Equation (4). Through mathematical transformations, it becomes evident that the second term in Equation (3) (or, equivalently, the term in Equation (4)) varies with angular speed. We, hence, anticipate increased error with the increase in motion speed.

(4) $$\frac{d\emptyset \left(\theta \right)}{dt}=\frac{d\emptyset \left(\theta \right)}{d\theta }\frac{d\theta }{dt}\;$$

### 2.2. Simulation Analysis 

Referring to Figure 2, the human legs (leg 1 and leg 2) are modeled as cylinders, each 4.4 cm in radius and 50 cm in length. We purposely select a canonical tissue-emulating phantom at this stage of the design for simplicity purposes. Referring to the sensor configuration in Figure 1, *Tx*1 and *Rx*1 are conformally placed on leg 1 while *Tx*2 and *Rx*2 are similarily positioned on leg 2. Loops on each leg are placed symmetrically around the joint, with a distance (d) of 10 cm between each pair of loops. The loops themselves are modeled as copper wire with an inner radius of 4 cm and a wire diameter of 0.0254 cm. To approximate the average distance between an adult’s legs, the gap (g) between the centers of the two cylinders is set at 20 cm. A lumped capacitor (C) of 100 pF is introduced to resnonate the loops at 34 MHz. The variables *θ_f_*_1_ and *θ_f_*_2_ denote the sagittal flexion angles of leg 1 and 2, respectively.

Simulations are conducted for the legs flexing from 0 to 90 degrees in increments of 10 degrees. This results in 100 (i.e., 10 × 10) distinct bilateral angular states for simulation. We aim to evaluate the transmission coefficients for the same leg (|S_21_| and |S_43_|, per loop numbering in Figure 2) across these states. Of course, given the symmetry of the setup, we can study the results from one leg only to corroborate the hypothesis of Section 2.1.2. For instance, considering leg 1, we are particularly interested in whether the transmission coefficient |S_21_| remains consistent when *θ_f_*_1_ is fixed while *θ_f_*_2_ varies from 0 to 90 degrees. All simulations are performed using CST Studio Suite 2021 and the frequency domain solver with tetrahedral meshing.

### 2.3. Experimental Analysis

#### 2.3.1. Sensor Fabrication Using Polymer-Embedded E-Threads 

The loop sensor design is exported using a scalable vector graphics (SVG) software (Inkscape, Ver 1.3.2) and digitized into a running stitch pattern using the Inkstitch plug-in function. The conductive material, namely a 40-filament Liberate e-thread, is embroidered onto regular fabric using a Brother Duetta 4500D (Aichi, Japan) automated embroidery machine. Lumped capacitors (100 pF) are soldered directly on each of the loops. Though SMA connectors could be soldered in a similar fashion, this would result in poor stability at the soldering point. This issue has been shown to be particularly problematic for tests with multiple repeating cycles. 

To address this limitation, we utilized a flexible polymer (namely polydimethylsiloxane, PDMS) to stabilize the SMA connector. To ensure no adverse impact on the sensor performance, we chose the SYLGARD 184 silicone elastomer which has a relative permeability of 1. All materials selected to implement the sensor are safe. PDMS that serves to encapsulate the sensor is a well-known biocompatible material, while the exterior coating of the e-thread is silver that is also known to be skin-friendly. 

**Figure 3:**
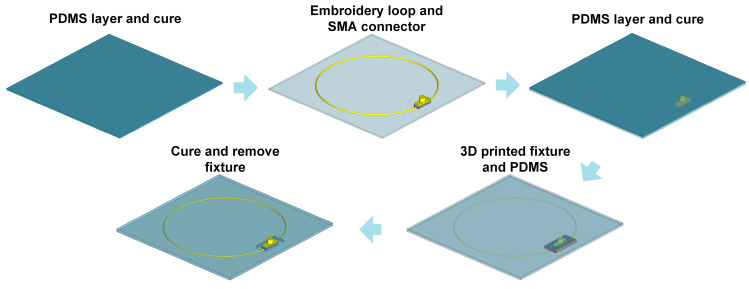
Process flow for manufacturing the polymer-embedded e-thread-based loops.

**Figure 4:**
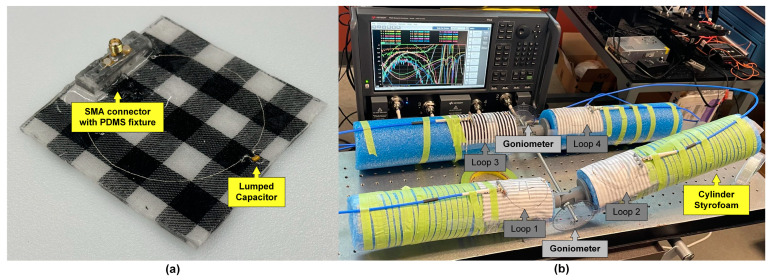
(**a**) Polymer-embedded e-thread-based loop sensor, (**b**) phantom-based experimental setup for bilateral knee flexion angle monitoring.

The detailed fabrication process is described in Figure 3. Specifically, the fabrication process is executed in layers, starting from the bottom. PDMS solution is poured into the mold and cured in an oven at 70 °C for 30 min. Next, the embroidered loop with the SMA connector is placed flatly on the first PDMS layer, using a small amount of PDMS solution. More PDMS solution is then added as the top layer and cured again at 70 °C for 30 min. The subsequent step involves firmly adhering the connector to the PDMS layer for added stability. For this, a 3D-printed hollow rectangular mold is placed on the bottom layer, with PDMS injected with a syringe. The final curing stage involves heating the assembly at 70 °C for 1 h (considering its thickness), followed by removal of the 3D-printed fixture. An example fabricated prototype is shown in Figure 4a. 

#### 2.3.2. Phantom-Based Experimental Setup

Given the inter-subject variability associated with testing on human subjects, canonical (in this case, cylindrical) phantoms are utilized as a first step of validation to generalize the findings. That is, phantom testing is a necessary preliminary step of validation prior to testing on human subject participants.

Referring to Figure 4b, the experimental setup utilizes Styrofoam limbs connected by a 3D-printed joint to create flexion and extension movements. The use of a Styrofoam limb has been explained in our previous work [36] and is an accurate representation of biological tissues in the deep inductive regime. A rod is employed to maintain the relative distance between the two legs. Goniometers are attached on both legs to allow for easy reading of the flexion angles. Fabric-based loop sensors (shown in Figure 4a with fabrication details provided in Section 2.3.1) are placed conformally on the cylindrical limbs. 

All dimensions match those described in Section 2.2. Similarly, in alignment with the simulation parameters, the flexion angles *θ_f_*_1_ and *θ_f_*_2_ vary from 0 to 90 degrees in 10-degree increments, to empower 100 angular states. The four loops are connected to a four-port network analyzer (PNA-X N5222B, Keysight Technologies, Santa Rosa, CA, USA) to measure the four-port S-parameters for each angular state at once.

#### 2.3.3. Human Subject Experimental Setup 

**Figure 5:**
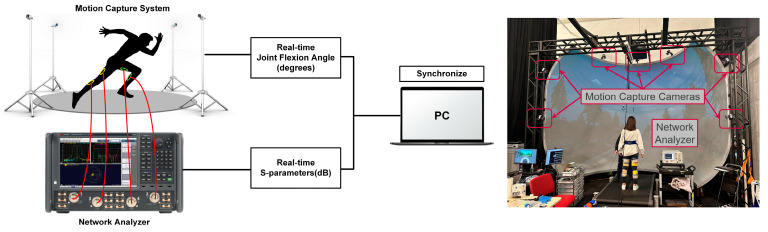
Experimental setup used for bilateral flexion angle monitoring on a human subject.

Referring to Figure 5, a 9-camera marker-based motion capture system (Mocap, Vicon Motion Systems Ltd., Oxford, UK) and a four-port network analyzer (PNL-X 5222B, Ver: A.14.20.04) were employed to collect data concurrently on a human subject. The four loops (per Figure 2) were placed conformally on the subject’s two legs using self-adhesive tape to prevent movement during rapid motions. They were connected to the network analyzer via coaxial cables connected to their SMA ports. Loop 1 and loop 2 were placed on the left leg while loop 3 and loop 4 were placed on the right leg. As a proof-of-concept, one human subject was recruited to participate in the study. The participant wore a 6 degrees-of-freedom lower body marker set for calculating bilateral joint flexion angles during four different types of motion on a treadmill for 30 s each: (a) slow flexion and extension across the range of motion, (b) walking, (c) brisk walking, and (d) running. All four activities were repeated three (3) times for both the right and the left leg. The participant provided Institutional Review Board (IRB)-approved written informed consent. 

The MoCap system recorded bilateral knee flexion angles (*θ_f_*_1_ for the left leg and *θ_f_*_2_ for the right leg) as “gold-standard”, while the network analyzer captured the four port S-parameters. A crucial step was to synchronize the time stamps for these two datasets, which we achieved through peak-to-peak alignment for all motions. Since we could not ensure that both datasets start exactly at the 0-s mark, we selected the time window from 5 to 25 s to account for any time shifts due to lack of synchronization. Data post-processing was performed next. Specifically, the first step is calibration, which involves establishing a one-to-one relationship between the transmission coefficients (|S_21_| and |S_43_|) and flexion angle for each leg. This is based on data from the slow, full flexion and extension motions. We choose this motion for two reasons: (a) it encompasses the full range of angles achievable for every motion in our experiments, (b) slower motions help minimize noise, including sensor displacement and motional EMF noise as will be explained in detail in Section 2.1.3. Calibration is unique to each leg and is a necessary step before each experiment since the placement of the loops determines the calibration result. With the calibration curves at hand, we can estimate the flexion angle values from the collected transmission coefficients (|S_21_| and |S_43_|). These estimated angle results are then compared with the “gold-standard” angles obtained from the MoCap system.

The purpose of this first-ever attempt to validate the sensor on human subjects is to demonstrate the ability to translate our phantom results on real anatomies, during dynamic motion. Having ensured feasibility on human subjects through this study, future research will focus on expanding upon the sample size and analyzing statistical aspects.

## 3. Results

### 3.1. Simulations and Phantom-Based Experiments 

#### 3.1.1. Sensor Performance

**Figure 6:**
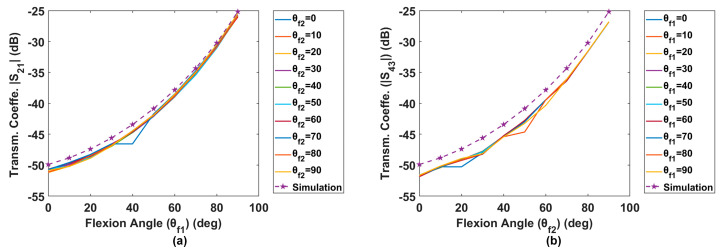
Simulation and phantom experimental results: (**a**) flexion curves of leg 1 (|S_21_|vs. *θ_f_*_1_) for different *θ_f_*_2_, (**b**) flexion curves of leg 2 (|S_43_|vs. *θ_f_*_2_) for different *θ_f_*_1_.

In Figure 6, simulation and phantom experimental results at the desired frequency (34 MHz) are shown together for comparison. Figure 6a illustrates the relationship between |S_21_| and flexion angle of leg 1 (*θ_f_*_1_) for varying flexion angle of leg 2 (*θ_f_*_2_). Figure 6b represents the symmetrical relationship between |S_43_|and flexion angle of leg 2 (*θ_f_*_2_) for varying flexion angle of leg 1(*θ_f_*_1_). 

In our discussion, we focus on leg 1 as the results are expected to be symmetrical. Specifically, as discussed in Section 2.1.2, our aim is to determine whether the flexion angle of leg 2 (*θ_f_*_2_) influences the |S_21_| value for different values of *θ_f_*_1_. In Figure 6a, there should be 10 simulation trials for different *θ_f_*_2_ values. Nevertheless, we have plotted only one because they all perfectly overlap, indicating that *θ_f_*_2_ does not affect the estimation of *θ_f1_* in the simulation. The experimental results align closely with the simulation, showing that all trials for different *θ_f_*_2_ values overlap, except for minor errors. Similarly, Figure 6b also provides strong evidence to support our hypothesis that the two legs’ transmission coefficients (and, hence, flexion angle estimations) are decoupled and do not interfere with each other.

#### 3.1.2. Tolerance to Noise Analysis 

Per Section 2.1.3, we expect the sensor to be more prone to noise at smaller angles where the calibration curve is relatively flatter. Indeed, as demonstrated by the simulation results of Figure 6, a minor error of 1.1 dB (from −49.9 dB to −48.8 dB) in |S_21_|at a small flexion angle (e.g., 0°) will lead to a 10° estimation error. Conversely, at a large flexion angle (e.g., 80°), a more substantial fluctuation of 5.1 dB in |S_21_| (from −30.2 dB to −25.1 dB) results in the same magnitude of angle estimation error (10°). This indicates that the system’s tolerance for error at larger flexion angles is five times greater than that at smaller flexion angles.

### 3.2. Human Subject Experiments 

#### 3.2.1. Sensor Performance 

**Figure 7:**
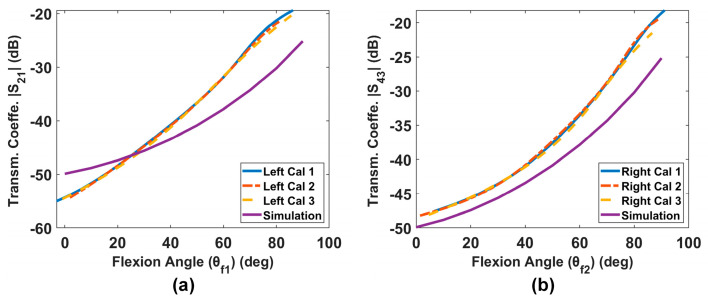
Calibration results from slow flexion and extension: (**a**) left leg, (**b**) right leg.

In this experimental setup, |S_21_| represents the transmission coefficient for the left leg while |S_43_| represents that for the right leg. Figure 7 shows the calibration curves (relationship between transmission coefficient and flexion angle) for both legs and compares these with the corresponding simulation result. Notably, the three calibration curves are almost identical, indicating that they can be reliably used to estimate angles from S-parameter measurements. The calibration process is crucial, as evident from the significant offset observed when comparing the experimental calibration curves with the simulation results. Also, it is important to note that the calibration for each leg differs substantially due to the placement of the loops.

**Figure 8:**
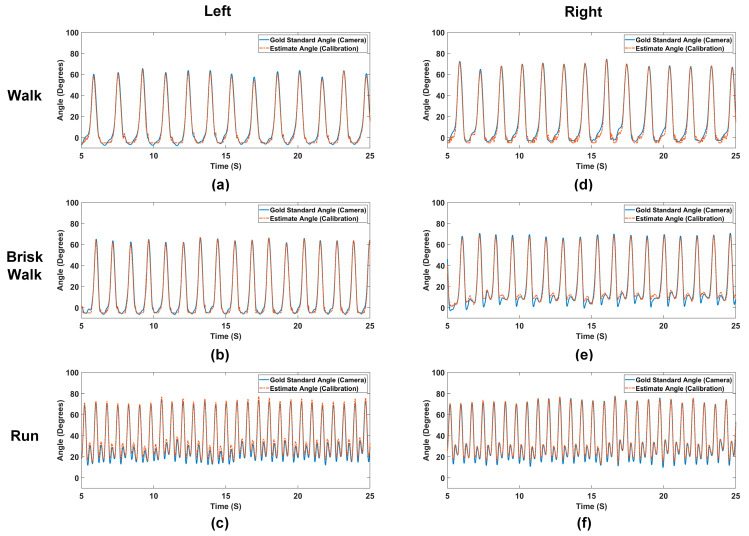
Representative real-time comparisons between the flexion angles captured by the sensor and MoCap system for 20 s during (**a**) walking, (**b**) brisk walking, and (**c**) running for the left leg; and (**d**) walking, (**e**) brisk walking, and (**f**) running for the right leg.

Figure 8 illustrates the results from representative motions of the participant over a 20-s duration. Results from other trials are very similar. As previously discussed, the transmission coefficients for both legs correspond to joint flexion angles. Utilizing the calibration curves we previously derived, we can estimate the flexion angle using the proposed sensor. Notably, the estimated angles (dashed red lines in Figure 8) demonstrate excellent agreement with the “gold-standard” angles (solid blue lines in Figure 8) for all three motions. To quantify this performance, we calculated the root mean square error (RMSE) and the coefficient of determination (R^2^) for all trials. Results are summarized in Table 2. According to Table 2, the RMSE for most trials is lower than 3 degrees, and the R² value is higher than 0.98, indicating a high level of accuracy between the estimations and the “gold standard”.

#### 3.2.2. Error Analysis 

Conducting an error analysis is critical for understanding the sensor’s operation and improving its design in the future. From Figure 8a,d, it is apparent that errors occur predominantly at small angles, approximately from the minimum to 10 degrees. As detailed in Section 2.1.3, the primary reason for this error is the high sensitivity of the estimated angle to slight variations in the transmission coefficient at smaller angles, where the calibration curve tends to flatten (see Figure 6 and Figure 7). 

Assessing the error performance across different angles and motions is also critical. To this end, we propose to analyze the RMSE across various angle intervals. This involves dividing the flexion angle range into intervals with a specific step width, such as 1 degree. We then find all the “gold standard” angles within that interval and their corresponding estimated angles. The final step involves calculating the RMSE for each interval, which can help better visualize the error across different angles and validate our theoretical analysis of Section 2.1.3. 

**Figure 9:**
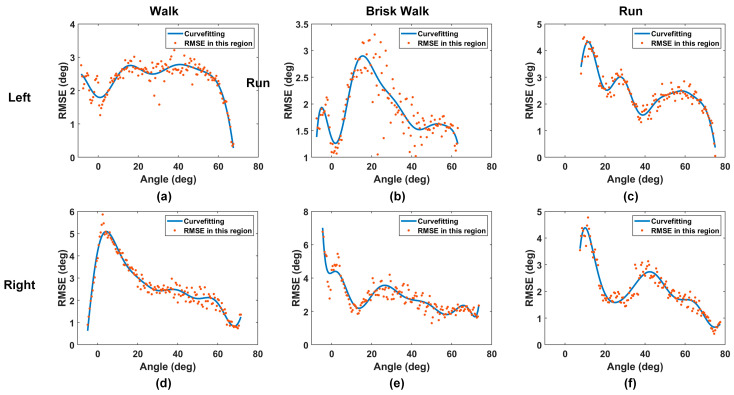
Representative interval RMSE and curve fitting result for 20 s during (**a**) walking, (**b**) brisk walking, and (**c**) running for the left leg; and (**d**) walking, (**e**) brisk walking and (**f**) running for the right leg.

Figure 9 illustrates the error performance across different angle intervals. A curve fitting method is applied to the discrete data points to eliminate potential noise and to elucidate clear trends. Focusing initially on the left leg, we observe from Figure 9a that, for walking trials, errors primarily occur within the 20–50 degree range. For brisk walking trials in Figure 9b, the error concentrates in the 10–40 degree range. Referring to Figure 9c, the error has several peaks (15 degrees, 30 degrees, and 50–60 degrees) for running trials. As seen in Figure 9d–f, the right leg demonstrates different error patterns: most errors are centered at small angles while some smaller peaks still occur around 30 degrees and 45 degrees for the brisk walking and running trials.

The prevalence of errors at small angles can be attributed to the flat calibration curve in this region, making the system vulnerable to loop movements and small changes in transmission coefficients. This phenomenon is more obvious in Figure 9d,e during walking and brisk walking, where errors at small angles can reach up to 5 degrees, adversely affecting the overall RMSE for the trial. 

We are also interested in why there were several peaks of errors for the brisk walking and running trails. These peaks are intriguing and appear to coincide with periods of high angular speed during flexion and extension. This can be explained by the presence of motion EMF, as detailed in Section 2.1.3. For both the walking and brisk walking trials, a single peak is observed in the error pattern, corresponding to the point where the angular speed reaches its maximum during the motion. On the other hand, in the running trials of Figure 8c,e, there are small angle fluctuations around 10–30 degrees. These fluctuations result in significantly high angular speed in that region, which explains the several peaks for the running trials in Figure 9c,f. 

In summary, the RMSE for walking and brisk walking is primarily attributed to the flatness of the calibration curve at small angles, whereas the RMSE for running is influenced by the large angular speeds. Overall, the RMSE for all trials is approximately less than 3 degrees with R^2^ value being greater than 0.98. These metrics demonstrate the high accuracy of our proposed sensors in various motion scenarios.

## 4. Discussion

In this paper, we proposed a wearable loop sensor designed to seamlessly monitor bilateral human flexion angles. A calibration and postprocessing method were reported to fuse and analyze data collected from the loop-based sensor and a “gold-standard” camera system used for validation. We conducted both simulation and phantom-based experiments to demonstrate feasibility, complemented by experiments on a human subject where new proposed calibration and synchronization methods were employed to achieve accurate estimations. Advances in science include:

(a) A wearable loop-based sensor configuration for bilateral knee flexion monitoring. Unlike previous studies that were limited to phantom models and discrete-time angles, our work represents the first real-time application of a bilateral loop sensor on the human body.
(b) A new fabrication process that enhances the stability of the connection between the loops and the SMA connector used for signal transmission/reception. Notably, when tested on a human subject, the integrated system exhibits an overall RMSE of less than 3 degrees, with R^2^ value close to 1, indicating excellent congruence with “gold-standard” results. These results support the 5° accuracy suggested by the American Medical Association for kinematic analysis in a clinical context [4] and surpass the state-of-the-art RMSE performance as summarized in Table 1.
(c) An analysis of the sensor’s sources of error with a goal to further optimize the design in the future. Notably, our error analysis went beyond the evaluation of the overall error in a single trial; it also examined errors across different angular intervals. This approach led to the insight that motional EMF contributes to larger errors at high angular speeds, such as during running trails. Another major source of error was attributed to the flatness of the calibration curve at small angular values, where minor fluctuations in loop displacement can result in substantial errors.

Despite the promising results, we would like to emphasize the limitations of this particular study. Specifically:

- Tests were reported on one human subject. This small sample is similar to several other studies performed in the literature during the first stages of wearable sensor validation [27,28,31,32,33]. It is herewith justified by our goal to study how the sensor system performs over the course of time and for different activities as relying upon a certain calibration curve. Additional participants would imply additional calibration curves (one for each participant) which is outside the scope of this particular work but will be explored in the future as critical for statistical validation.
- Three trials were performed for each type of activity. Given the consistency in sensor performance among different trials, we expect similar behavior for additional trials as well. As an example, two additional trials were performed for the brisk walking activity (as an intermediate case between the walking and running activities), which only slightly modified the average RMSE from 2.10° for the left leg and 3.01° for the right leg in Table 1 to 2.09° for the left leg and 3.15° for the right leg.
- The loop sensors are not yet fully wearable. That is, cables are used to connect to a network analyzer. In the future, we plan to develop circuits boards for the wireless collection of transmission coefficient measurements without the need for a network analyzer. This advancement would require careful consideration of mutual coupling as the circuit board would excite the two *Tx* loops simultaneously, unlike the network analyzer that excites one *Tx* loop at a time.
- To minimize noise, we relied on self-adhesive bands to secure the loops firmly on the legs. Higher levels of noise would likely be present if we were to sew the loops directly on the cloth without any stabilization.

Looking forward, we envision further miniaturization of the sensor using a spiral shape. Moreover, we are considering the application of machine learning to generalize the calibration curve, eliminating the need for calibration before each experiment. To reduce/eliminate the sources of noise, we can: (a) optimize the sensor design such that the calibration curve slope becomes steeper, (b) explore feedback mechanisms and adaptive features to overcome external noise (e.g., changes in the calibration curve in response to loop displacement), and (c) identify the exact relationship between EMF-induced error and angular velocity to compensate for motion EMF. Ultimately, our goal is to integrate real-time bilateral angle measurement into regular clothing in a simple, unobtrusive manner, paving the way for a wide range of clinical applications for our sensor technology. 

## Figures and Tables

**Table 2:** RMSE and R^2^ values between estimated result and “gold standard” for all trials.

	Left Leg						Right Leg					
Trial	Walking		Brisk Walking		Running		Walking		Brisk Walking		Running	
	RMSE	R^2^	RMSE	R^2^	RMSE	R^2^	RMSE	R^2^	RMSE	R^2^	RMSE	R^2^
1	2.22°	0.9946	2.48°	0.9947	2.37°	0.9838	3.30°	0.9918	2.93°	0.9871	2.00°	0.9893
2	2.19°	0.9932	1.84°	0.9956	3.23°	0.9823	2.88°	0.9933	3.08°	0.9869	2.08°	0.9894
3	1.96°	0.9948	1.99°	0.9952	3.14°	0.9853	2.61°	0.9935	3.02°	0.9862	2.31°	0.9883
**Avg**	**2.12**° **± 0.16**°	**0.9942 ± 0.0010**	**2.10**° **± 0.38**°	**0.9952 ± 0.0005**	**2.91**° **± 0.54**°	**0.9838 ± 0.0015**	**2.93**° **± 0.37**°	**0.9929 ± 0.0011**	**3.01**° **± 0.08**°	**0.9867 ± 0.0005**	**2.13**° **± 0.18**°	**0.9867 ± 0.0007**

## Data Availability

Data is available upon reasonable request.
